# Climatic Drivers of the Complex Phenology of the Mediterranean Semi-Deciduous Shrub *Phlomis fruticosa* Based on Satellite-Derived EVI

**DOI:** 10.3390/plants11050584

**Published:** 2022-02-22

**Authors:** Aris Kyparissis, Efi Levizou

**Affiliations:** Department of Agriculture Crop Production and Rural Environment, University of Thessaly, Fytokou Str., 384 46 Volos, Greece; elevizou@uth.gr

**Keywords:** remote sensing, MODIS, enhanced vegetation index, temperature, precipitation, rain days, inter-annual variability, time-series, machine learning, climate change

## Abstract

A 21-year Enhanced Vegetation Index (EVI) time-series produced from MODIS satellite images was used to study the complex phenological cycle of the drought semi-deciduous shrub *Phlomis fruticosa* and additionally to identify and compare phenological events between two Mediterranean sites with different microclimates. In the more xeric Araxos site, spring leaf fall starts earlier, autumn revival occurs later, and the dry period is longer, compared with the more favorable Louros site. Accordingly, the control of climatic factors on phenological events was examined and found that the Araxos site is mostly influenced by rain related events while Louros site by both rain and temperature. Spring phenological events showed significant shifts at a rate of 1–4.9 days per year in Araxos, which were positively related to trends for decreasing spring precipitation and increasing summer temperature. Furthermore, the climatic control on the inter-annual EVI fluctuation was examined through multiple linear regression and machine learning approaches. For both sites, temperature during the previous 2–3 months and rain days of the previous 3 months were identified as the main drivers of the EVI profile. Our results emphasize the importance of focusing on a single species and small-spatial-scale information in connecting vegetation responses to the climate crisis.

## 1. Introduction

Vegetation response to climate variability is becoming increasingly important, especially under the frame of the ongoing global climate change [1,2,3]. Our understanding of vegetation function, its interactions with the climate, the key controlling mechanisms, and its vulnerability to climate change are far from complete. Evidently, understanding climatic influences on processes and interactions enables the prediction of changes under different climatic scenarios [4,5].

The most consistent results of climate change experiments are the species-specific responses. Many experiments that have been conducted worldwide, including manipulations of the CO_2_ and UV-B environment, temperature rise, and N deposition, have manifested that plant species differ in their sensitivity to damage and their morphological, biochemical, and physiological responses to altered environmental factors [6,7]. The exact position held by a certain species in the sensitivity-tolerance continuum, as well as its specific responses, could cascade upwards to alter community and ecosystem composition and structure through changes in the competitive balance between species [8,9]. Additionally, the proposed conceptual frameworks for analysis of the species and ecosystem response to changing climate underline the importance of thresholds for interpreting experimental results and predicting effects [10]. Rapid, nonlinear changes in some plant processes or responses can be triggered by even small differences in environmental conditions if threshold values are exceeded. This evidence stresses the importance of studies focusing on direct and indirect effects of environmental change on plant species and not only on large formations and ecosystem-level spatial scales.

Plant phenology is considered an important factor that mediates vegetation and climate relationships, through affecting a diverse set of processes [2]. Phenology is not merely the succession of recurrent biological events in the plant’s lifecycle, but it also greatly relates to plant activity, since different phenophases affect plant function and resource allocation patterns [11]. The phenology–climate feedbacks are bi-directional. At one end is the climate impact on the timing and duration of phenological events [3,12]. At the other end is the influence of phenological events and, moreover, transitions on vegetation feedbacks to the microclimate, i.e., humidity, temperature, wind speed, as well as soil moisture and topsoil temperature [2]. Scaling up at the community and ecosystem level, phenology influences processes and mechanisms such as water, CO_2_, and energy fluxes which feedback to large-scale vegetation–atmosphere interactions. The well-established sensitivity of phenology to year-to-year variability in climate could also serve as an indicator of the long-term biological impacts of climate change on terrestrial ecosystems [13,14].

Remotely sensed data proved to be a valuable tool in coupling climate and vegetation distribution/performance at large spatial and temporal scales. As a result, the objective of many studies was the assessment of the effects of certain environmental factors on remote-sensing-derived vegetation parameters [15,16,17]. A common feature in most of the above-mentioned research efforts is the large spatial scale used, i.e., regional, continental or global, exploiting satellite-derived simultaneous estimates of ecosystem function over wide areas. Indeed, remote sensing of vegetation offers a promising and urgently needed assessment of ecosystem function at a spatial scale that is comparable with the extent of human-caused environmental change. However, information on specific species performance, which is possibly incorporated in remote sensed data, is rather lost in the inevitably vague picture given by large spatial-scale studies [18]. Ecophysiological field surveys could address this issue, but because of laborious and time-consuming measurements, they have the disadvantage of temporal and spatial limitations. Alternatively, satellite imagery in the context of studying a particular species’ behavior, i.e., at small spatial scale, may render an accurate picture of species responses to natural climate variability, as well as climate change [19,20].

The focus on species and use of satellite data to study species-level responses, from a phenological and especially an eco-physiological point of view, to climate forcing has an essential prerequisite: strong correspondence with ground-measured plant processes or features [21,22]. Indeed, established relationships between ground-determined characteristics and their satellite-derived surrogates in terms of vegetation indices allow for an explicit physiological meaning to the latter. This in turn permits understanding, monitoring, and explaining species behavior, as well as identifying broad patterns in space and time, including a species’ relationship with environmental determinants. Collectively, established correlations enable exploiting the advantages of remote sensed data, i.e., large spatial and temporal scales, with direct reference to species phenological/physiological characteristics. The above-mentioned benefits justify the intensive research efforts of the last two decades devoted in establishing such correlations [22,23,24,25].

The Enhanced Vegetation Index (EVI) has been shown to be well correlated with LAI, biomass, canopy cover, and the fraction of absorbed photosynthetically active radiation [26,27], and is therefore useful for monitoring seasonal, inter-annual, and long-term variation of the vegetation structure and function [28]. EVI has been used instead of the Normalized Difference Vegetation Index (NDVI) because it reduces sensitivity to soil and atmospheric effects and remains sensitive to variation in canopy density where NDVI becomes saturated [29,30,31]. Given these characteristics, modelers have begun using EVI data to predict net primary production in ecosystem modelling applications [24,29].

Under the above-described framework, a 21-year EVI time-series is used in the present study to assess climatic effects on the growth cycle of the drought semi-deciduous Mediterranean shrub *Phlomis fruticosa*. *P. fruticosa* was chosen for the following reasons: (a) it is a typical drought shrub of Mediterranean ecosystems and, moreover is considered a key species of the garrigue vegetation which dominates the most xeric parts of the Mediterranean basin [32]; (b) we consider this species an ecological indicator of overgrazed ecosystems in which it forms large, continuous, and undisturbed stands, exclusively covered by this particular species, because it is not eatable by major farm animals (sheep, goats); (c) it has a multi-phase intra-annual growth cycle, with distinct phases being possible targets to climatic effects, which makes *P. fruticosa* a good model plant for satellite-derived phenology studies; (d) its growth cycle has been extensively studied through eco-physiological field measurements [33,34]; thus, there are established relationships between growth/eco-physiological parameters and satellite indices [22,25]; (e) the plasticity and adaptability of *P. fruticosa*, although established by field eco-physiological measurements have not been validated in large space and time scales, as those provided by satellite imagery.

For the purposes of the present study, two distant *P. fruticosa* ecosystems, with differences in climatic characteristics were chosen. The aims were (a) to depict the complex phenological cycle of this species through satellite-derived EVI and extract metrics that analyze the phenological events and transitions, (b) to determine the climatic drivers that influence the phenophases and their possible differences between the two sites, and (c) to identify trends for change, which could further be used as diagnostic and prognostic tool for climate crisis effects.

## 2. Materials and Methods

### 2.1. Study Sites

**Figure 1:**
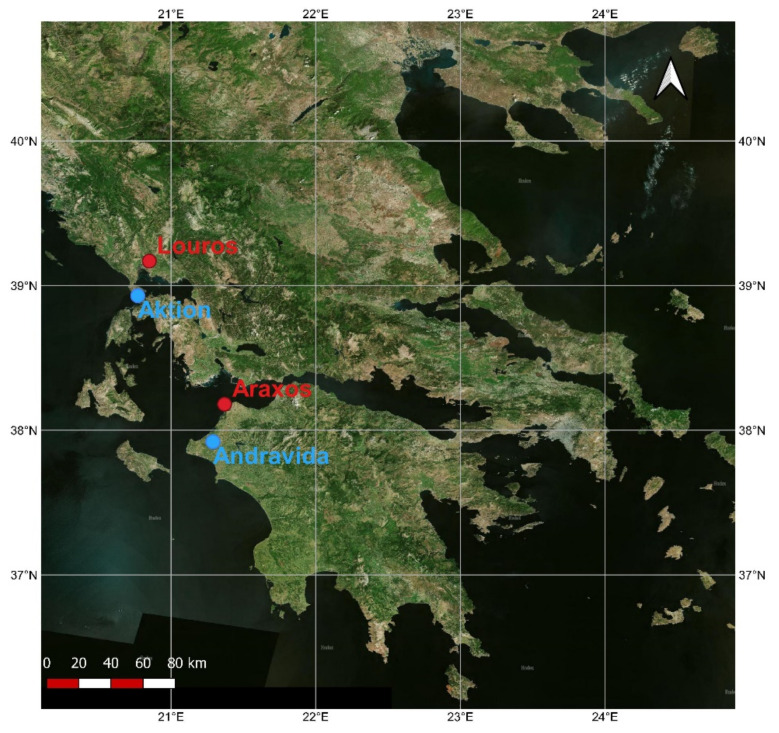
Map of Greece with the two study sites indicated with red dots and the locations of the meteorological stations with blue dots.

Two ecosystems dominated by *P. fruticosa* with different climatic characteristics were chosen in order to study possible differences in climate control and plant responses (Figure 1): (a) Araxos area, the southern one (NW Peloponessos, Greece, 38.18° N, 21.37° E), characterized by a prolonged summer stress period where high temperature co-exists with a severe water shortage and (b) Louros area, the northern one (Epirus, Greece, 39.17° N, 20.85° E), with more favorable temperature and water availability conditions for plant growth.

### 2.2. Meteorological Data

**Figure 2:**
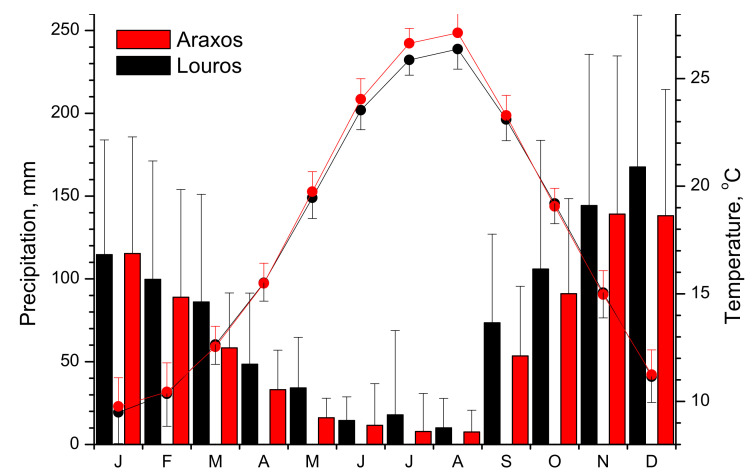
Annual profile of the average total monthly precipitation (bars) and the average monthly temperature (points) for the 21-year study period (2000–2020) for Araxos and Louros study sites.

Meteorological data (average daily temperature and daily precipitation) of the 21-year study period (2000–2020) for Araxos site were recorded by a meteorological station situated in Andravida, about 29 km from the study area, while for Louros site, in Aktion airport, about 28 km from the study site. Data were downloaded from U.S. National Oceanic and Atmospheric Administration (NOAA) National Climatic Data Center (NCDC, www.ncdc.noaa.gov, accessed on 1 March 2021). In Figure 2, the annual profile of the average total monthly precipitation and the average monthly temperature for the 21-year study period is presented for both study sites. Increased rain amounts in Louros site throughout the year and a slightly lower temperature during the stressful summer period are evident (average temperature of June to August 25.93 ± 0.62 °C for Araxos and 25.25 ± 0.63 °C for Louros). For Araxos, the average annual temperature is 17.86 ± 0.35 °C and total annual precipitation 759.5 ± 151.8 mm, while for Louros, the temperature and precipitation were 17.64 ± 0.42 °C and 919.3 ± 217.2 mm, respectively.

### 2.3. Species Description

*Phlomis fruticosa* is a dimorphic, semi-deciduous shrub of the eastern Mediterranean areas. It bears two kinds of leaves—winter and summer ones—with different biochemical and structural characteristics [30]. Winter leaves and summer leaves of the previous growing season are massively shed during mid to late spring, resulting in a decrease in Leaf Area Index (LAI). Hereinafter, we refer to this event as spring drop. During the summer dry period, plants bear summer leaves developed during spring, which are smaller than winter leaves and have lower chlorophyll content. After the onset of the autumn rainy period, summer leaves absorb water rapidly (within days) and increase their area, while they alter their biochemical characteristics, including chlorophyll content increase. Additionally, new winter leaves with high chlorophyll content appear during November. Hereinafter, we refer to this event as autumn revival. The transformation of summer leaves and the appearance of winter leaves during autumn result in an increase in LAI, which remains almost stable during winter, until next spring. Even though this phenological/physiological cycle is repeated every year, the extent and/or the exact date for each particular phenological event seem to depend on the microenvironmental conditions [31]. The main physiological advantage of the semi-deciduous habit is the decrease in the transpiring leaf area during the summer dry months, resulting in more efficient water economy.

### 2.4. Satellite Data

Τhe Enhanced Vegetation Index (EVI) was used in the present study. For the calculation of EVI, data from the Moderate Resolution Imaging Spectroradiometer (MODIS) onboard the Terra satellite (part of the NASA Earth Observing System) were used. MODIS scans the entire Earth surface every 1–2 days, acquiring data in 36 spectral bands. Out of the 36 spectral bands, 7 bands are designed for the study of vegetation and land surfaces: blue (459–479 nm), green (545–565 nm), red (620–670 nm), near infrared (NIR1: 841–875 nm, NIR2: 1230–1250 nm), and shortwave infrared (SWIR1: 1628–1652 nm, SWIR2: 2105–2155 nm). Several products with differences in spectral, spatial, and temporal resolution, as well as in correction levels are freely provided by the MODIS Land Science Team to users. In the present study, the geometrically and atmospherically corrected Surface Reflectance 8-Day L3 Global 500 m product (MOD09A1), available to the public from the US Geological Survey EROS Data Center (USGS EROS Center, http://eros.usgs.gov/, accessed on 1 March 2021), was used. EVI was calculated according to the equation:

(1) $$EVI=2.5\frac{R_{nir}-R_{red}}{R_{nir}+6R_{red}-7.5R_{blue}+1}$$

where *R_nir_* is reflectance between 841 and 875 nm, *R_red_* between 620 and 670 nm and *R_blue_* between 459 and 479 nm [35].

The MOD09A1 datasets (2000–2020), which have a 500 m spatial resolution and 8-day temporal resolution, were downloaded from the USGS EDC website using the geographical coordinates of each study site and 21 years EVI time-series were produced for 4 and 3 pixels for Araxos and Louros sites, respectively. These pixels were selected for each site after land observations and GPS recording, as being homogenous and dominated exclusively by *P. fruticosa*. The time-series of each pixel were corrected for erroneous values during cloudy dates or other reasons using the BISE (Best Index Slope Extraction) algorithm [32]. Accordingly, for each date the average of the corresponding pixels was calculated for each site and used for the construction of time-series over the 21-year study period. The time-series of the two sites were further smoothed using an adjusted Fast Fourier Transform [36]. Additionally, the average annual EVI profile for each site was calculated by averaging EVI values for the same 8-day period between years (Figure 4).

### 2.5. Phenology Metrics

The phenological cycle of *P. fruticosa*—as described above—may be depicted by the seasonal EVI fluctuation shown in Figure 2. High and rather stable values of EVI during winter (December to March), corresponding to high LAI and chlorophyll content values, are followed by the spring drop period (April to June). The steep reduction in EVI during that period corresponds to the massive loss of winter leaves and summer leaves of the previous year. During summer dry period (July to September), plants bear a small number (low LAI) of low chlorophyll content summer leaves (low and stable EVI values). The subsequent autumn revival period, coinciding with the onset of the autumn rainy period (October–November), is characterized by an abrupt rise of EVI, as a result of the “resurrection” of summer leaves (rapid increase in leaf area due to water absorption, accompanied by chlorophyll content increase) followed by the burst of new winter leaves at the end of autumn. This pattern is repeated every year in both study sites, but remarkable differences in parameters of spring drop, dry period and autumn revival may occur among sites and/or years.

**Figure 3:**
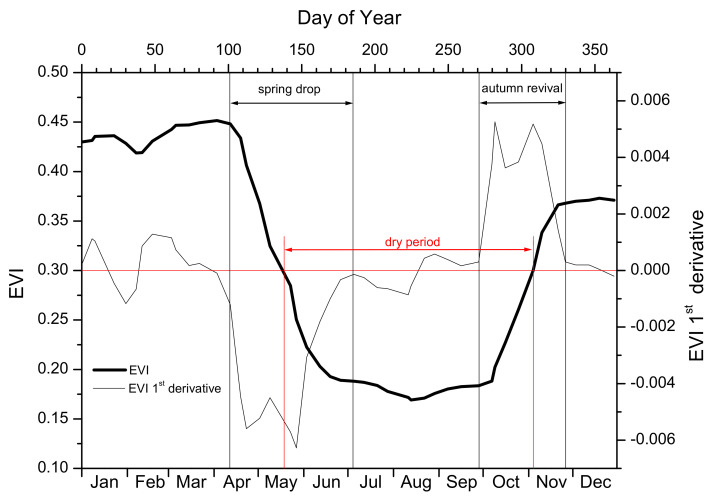
Typical profile of the annual fluctuation of EVI (thick line) and its 1st derivative (thin line). Red horizontal line corresponds to EVI = 0.3 and EVI 1st derivative = 0. Black vertical lines indicate the onset and end of the spring drop and the autumn revival period, when EVI 1st derivative departs or returns to zero values for onset and end correspondingly. Red vertical lines indicate the onset and end of the dry period, when EVI is lower than 0.3.

In order to quantify the phenological events described above (spring drop, dry period, and autumn revival), the parameters presented in Table 1 were calculated for each site and year. For the calculation of spring drop and autumn revival related parameters, the 1st derivative of the EVI curve was used. Day of Year for spring drop onset (SDO) and autumn revival onset (ARO) are determined when the 1st derivative departs from near zero values (Figure 3) and spring drop end (SDE) and autumn revival end (ARE) when the 1st derivative returns to near-zero values, during the spring and autumn EVI steep change period. The differences between SDE-SDO and ARE-ARO result in the spring drop duration (SDD) and autumn revival duration (ARD), respectively.

Concerning dry period parameterization, onset (DPO) and end (DPE) of the dry period were calculated according to the threshold method [37,38]. The EVI value of 0.3 was defined as the threshold for DPO and DPE and was chosen because it represents the midpoint between absolute maximum and absolute minimum (all 21 years) EVI values. Thus, DPO and DPE were quantified as the Day of Year at which EVI reaches or leaves 0.3, respectively, and the dry period duration as their difference (Figure 3).

Additionally, in an attempt to exploit all the information contained in the shape of EVI curve, annual maximum and minimum values and the date of their occurrence were also determined for each site and year (Table 1). Finally, mean monthly EVI was calculated and used as a surrogate of ecosystem dynamics in the assessment of climate control on growth features. All the above-mentioned extracted parameters were used as independent phenology metrics in the statistical analyses (see below) for the identification of the most influential climatic factor.

### 2.6. Statistical Analysis

The relationships between the above described phenological events and climatic parameters were assessed using Pearson correlations and stepwise multiple linear regressions. The examined climatic parameters concern total monthly precipitation (P), monthly sum of rainy days (RD) and mean monthly temperature (T), of different time intervals-concurrent and lagged-in relation to the corresponding phenological event. More specifically, phenological events were examined against the following combinations:

1. Average temperature, total precipitation, and total rain days of one to six consecutive months before each phenological event, e.g., for a phenological event occurring in October, total precipitation of October and September (two months combination).
2. Average temperature, total precipitation, and total rain days of one to five consecutive months with one to five months lag before the event, e.g., for a phenological event occurring in October, total precipitation of August and July (two months combination with two months lag time).

The first step was to perform a Pearson correlation analysis for each phenological event and the above-mentioned climatic parameters. Accordingly, the independent variables that exhibited the maximum correlations in each case were employed in multiple linear regression analyses with stepwise selection. Collinearity of predictor variables was automatically detected by the statistical software and subsequently dealt by omitting variables and re-running the regression analysis.

The influence of climatic parameters on inter-annual EVI variation was examined following two different regression analysis methods, i.e., multiple linear regression and random forest machine learning. As in the case of phenology and climate control, all combinations of the climatic parameters (precipitation, rain days, temperature) of various time intervals and time lags of consecutive months up to six months before the event (EVI of a particular month) were considered. Initially, a monthly step EVI time-series was produced for each site, by averaging the analytical time-series data for each month. For the first approach, the most significant climatic parameters were determined through single linear regressions between monthly EVI and climatic parameters. Accordingly, combinations of the most important parameters were examined through stepwise multiple linear regression. Analyses showed that a high regression coefficient was achieved with two climatic parameters, i.e., adding additional parameters did not significantly enhance the efficiency of the regression (data not shown). On the second approach, all climatic parameters were used in a random forest machine learning procedure. During this procedure, data were randomly split into a training set (64% of data), a validation set (16% of data) for performance optimization and a test set (20% of data) for assessment of performance efficiency. However, for an overall comparison of machine learning with multiple linear regression all data were used in Figure 8. The efficiency of the two approaches was assessed through the coefficient of determination (R^2^) and Root Mean Square Error (RMSE) of the predicted EVI against actual EVI values.

All statistical analyses were performed with JASP v.0.16 software (JASP Team 2021 Computer Software), which includes a machine learning module.

## 3. Results

### 3.1. Phenology

**Figure 4:**
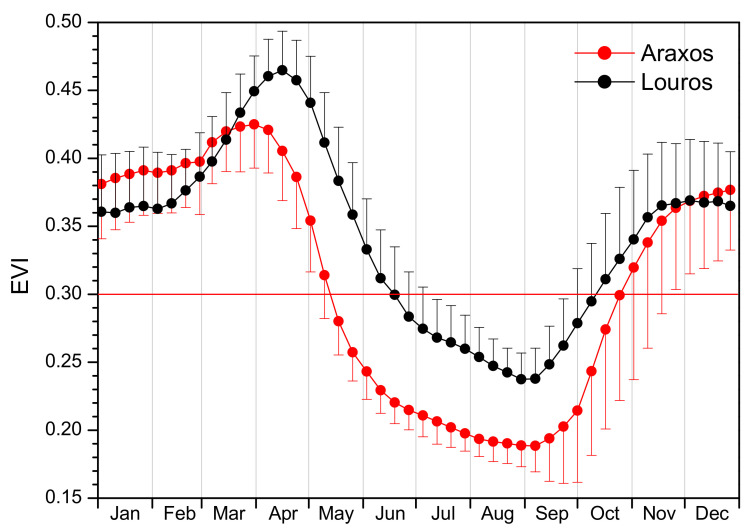
Annual EVI profile for the two study sites as average ± SD from the 21-year study period data. The red line marks EVI = 0.3, the value which was defined as the threshold for dry period onset and end.

In Figure 4, the annual EVI profile for the two study sites is presented as average ± SD from the 21-year study period data. In Araxos, spring drop as well as dry period (EVI < 0.3) starts earlier and dry period ends later in autumn, accompanied by a retarded autumn revival. Additionally, annual maximum and minimum EVI values appear higher for the Louros site compared with Araxos, possibly as a result of better physiological performance and/or higher shrub density under the more favorable conditions of Louros.

**Figure 5:**
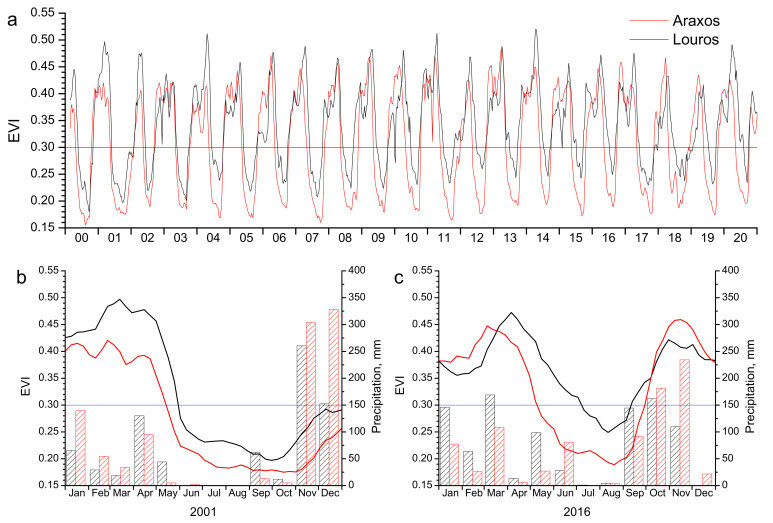
Interannual EVI fluctuation for the 21-year study period for the two study sites (**a**); EVI fluctuation and the corresponding precipitation profile during a dry (2001, **b**) and a wet (2016, **c**) year in the two study sites. The blue line marks the threshold for dry period onset and end (EVI = 0.3).

These general differences among sites are usually followed every year throughout the 21-year study period (Figure 5). Additionally, it is clear from Figure 5 that the annual profiles for both sites show strong differences from year to year. These interannual variations may be rather large, especially during summer periods, as seen by comparing the very dry summer of 2001 with the wet summer of 2016 (Figure 5).

**Figure 6:**
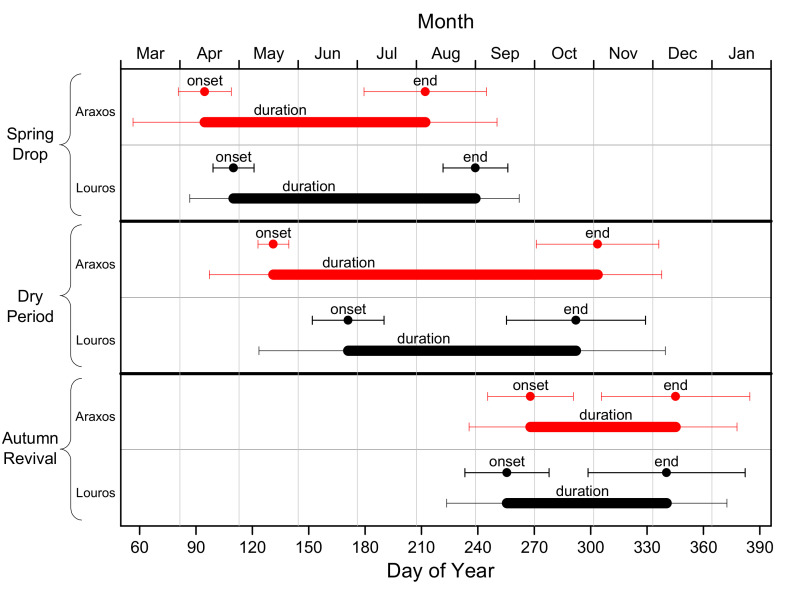
Graphical depiction of onset, duration, and end of the main phenological events (Spring Drop, Dry Period and Autumn Revival) for the two study sites. For all events, onset and end correspond to Day of the Year, whereas duration corresponds to number of days (data from Table 2).

In an attempt to reveal the detailed differences between sites, the phenological events described in Table 1, were determined for all years and sites and their average values are presented in Table 2, whereas the most important among them, i.e., events concerning spring drop, dry period, and autumn revival, are depicted in Figure 6.

As shown in Table 2, onset and end of spring drop occur 15 and 27 days earlier in Araxos compared with Louros, respectively, with both events showing statistically significant differences. Additionally, spring drop in Araxos occurs more rapidly, as indicated by the smallest duration, compared with Louros. Dry period starts 40 days earlier and finishes 11 days later in Araxos compared with Louros, resulting in a significantly longer dry period duration by 51 days. Concerning autumn revival, onset and end appear 13 and 5 days later, respectively, in Araxos, even though only onset is significant. However, as in the case of spring drop, autumn revival occurs more rapidly in Araxos (shorter duration), compared with Louros. Maximum and minimum EVI values are both significantly higher in the Louros site, where more favorable climatic conditions prevail. Accordingly, the date of maximum EVI appears 22 days earlier in Araxos, whereas no statistical difference is evident for the date of minimum EVI.

### 3.2. Phenology and Climatic Control

In order to account for climatic controls of phenology, average monthly temperature, total monthly precipitation and total monthly rain days of time windows relevant to each event and transition were examined. More specifically, phenological events were examined against climatic parameters of various time intervals and time lags up to six months before the event (see Section 2.6).

**Figure 7:**
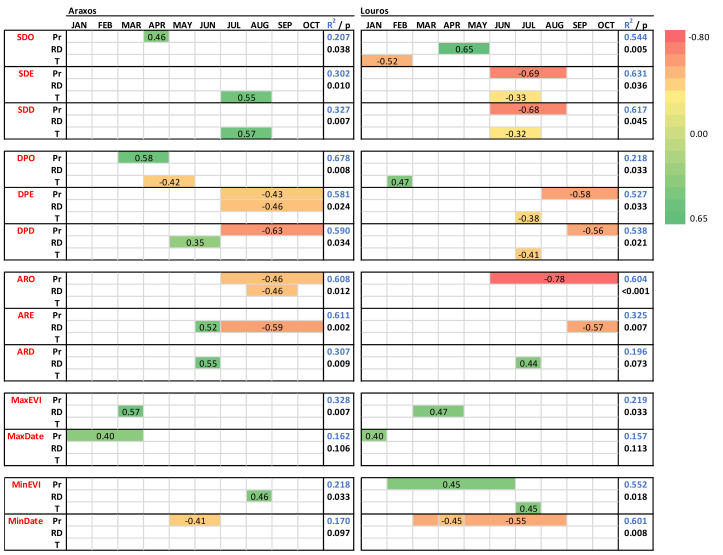
Climatic control on phenological events and transitions of *P. fruticosa* in the two sites as derived by single or multiple linear regressions. The phenological metrics are: Spring drop onset (SDO); spring drop end (SDE); spring drop duration (SDD); Dry period onset (DPO); dry period end (DPE); dry period duration (DPD); Autumn revival onset (ARO); autumn revival end (ARE), autumn revival duration (ARD); MaxEVI the maximum value of EVI; MaxDate, the date it is achieved; MinEVI the minimum value of EVI; MinDate, the date it is achieved. For each phenological event, the partial regression coefficient(s) of the most significant climatic variable(s) (precipitation (Pr), number of rain days (RD) and temperature (T)) of single or multiple months (top line) is presented in the corresponding colored horizontal lines, according to the chromatic scale appearing in the right. The regression coefficient (R^2^) of the model which includes the factors influencing each event and the corresponding level of significance (p) is presented in the right column of each site.

Spring drop onset for Araxos is influenced by precipitation of April (Figure 7), with more rain delaying SDO. Accordingly, Louros is similarly influenced by the rain days of April and March, whereas the temperature during January and February also plays a role; low temperature delays SDO, probably through sustaining higher soil water capacity.

In Araxos, where precipitation during summer months is minimal and with low interannual variability (July and August rainfall of 15 ± 33 mm), the main influential factor on SDE is temperature of July and August with high temperature delaying the SDE and resulting in lower minimum EVI values compared with Louros (Table 2). On the contrary, in the wetter and more variable Louros site (July and August rainfall of 29 ± 61 mm), SDE is mainly influenced by summer precipitation: the more it rains the earlier SDE appears and at higher minimum EVI value compared with Araxos.

Dry period onset for Araxos, is affected by the precipitation of March and April (higher precipitation delays onset) and the temperature of April and May (higher temperature advances onset). For Louros, DPO is mildly affected by the February temperature, with higher temperature delaying the event. DPE for Araxos is strongly affected by summer-early autumn rain, since both precipitation and rain days of July to October period influence the event, causing a delay at drier years. Similar effects of precipitation are evident for Louros, but for this site, the temperature in July also plays a minor role; a higher temperature results in earlier DPE and shorter duration.

Autumn revival onset is also strongly affected by rain (precipitation of July to October and rain days of August and September), with earlier onset during wetter years. A similar effect of rain is apparent for Louros, but only through precipitation (June to October). For both sites, ARE is affected by autumn rain days (July to October for Araxos and September to October for Louros). Nevertheless, a rather unreasonable effect of June rain days is evident in Araxos, where more rain days during June delay the ARE. However, this peculiar effect is also recorded for ARD for both sites (rain days of June for Araxos and July for Louros).

The value of maximum EVI seems to be positively affected by spring rain days for both sites (of March for Araxos and March–April for Louros). Winter precipitation affects the date in which the maximum EVI is achieved for both sites. More specifically, more rainfall during January to March for Araxos and January for Louros causes a delayed appearance of maximum EVI. Minimum EVI occurs during late August or early September in both sites. The rain days of August is the determinant of minimum EVI in Araxos, whereas precipitation over a longer period, February to June, positively affects the minimum EVI of Louros. Additionally, Louros seems to be affected by temperature of July, but in a rather unexpected way, since higher temperature results in higher EVI. Finally, for both sites the date that the minimum EVI appears is affected by the spring–summer precipitation (May–June for Araxos and March to August for Louros), with more rain transferring the date earlier.

Collectively, the 13 phenological events analyzed above are influenced mostly by rain related parameters for the Araxos site; more specifically 10 events by precipitation and/or rain days, 2 events by temperature and 1 event by both rain and temperature. On the contrary, both rain and temperature play crucial roles in Louros phenology, since 6 events are influenced by precipitation and/or rain days, 6 events by both rain and temperature, and 1 event by temperature.

### 3.3. Phenology and Climate Change

**Figure 8:**
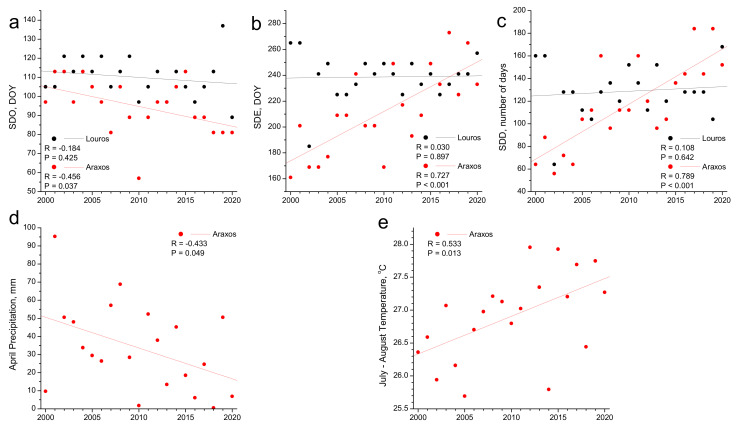
Interannual fluctuation of the spring drop related phenological events (dots) and their trends (lines) for the two study sites during the study period: (**a**) Spring drop onset (SDO,), (**b**) spring drop end (SDE), (**c**) spring drop duration (SDD. Interannual fluctuation of the main climatic parameters influencing the phenological events of the Araxos site (dots) and their trends (lines) during the study period: (**d**) April precipitation, (**e**) July–August temperature.

All phenological events examined above could be potentially related to the ongoing climate change. Our dataset of 21 years is long enough to permit the analysis of the trends of phenological events’ interannual fluctuation in the context of climate change. As shown in Figure 8, spring-drop-related events show significant trends for the Araxos site, but not for Louros, whereas no significant trends appear for the rest of the phenological events (data not shown). The trends appearing for Araxos seem to be explained by similar trends in the main climatic factors that these events are related to (Figure 8). SDO tends to commence earlier in the season by 1 day per year, whereas April precipitation—the main influential climatic parameter (Figure 7)—tends to decrease by 1.7 mm per year. Accordingly, SDE experiences a delay by 3.8 days per year and spring drop duration is elongated by 4.9 days per year. Both events are influenced by July–August temperature (Figure 7), which shows a similar trend, increasing by 0.06 °C per year during the study period (Figure 8).

### 3.4. EVI and Climatic Control

Since all phenological parameters are extracted from the EVI time-series, the influence of climatic parameters on EVI per se was examined as a final integrating step. To that purpose, as in the case of climate control on phenological events, all combinations of the climatic parameters (precipitation, rain days, temperature) of various time intervals and time lags of consecutive months up to six months before the event (EVI of a particular month) were considered through two regression analysis methods, i.e., multiple linear regression and random forest machine learning.

**Figure 9:**
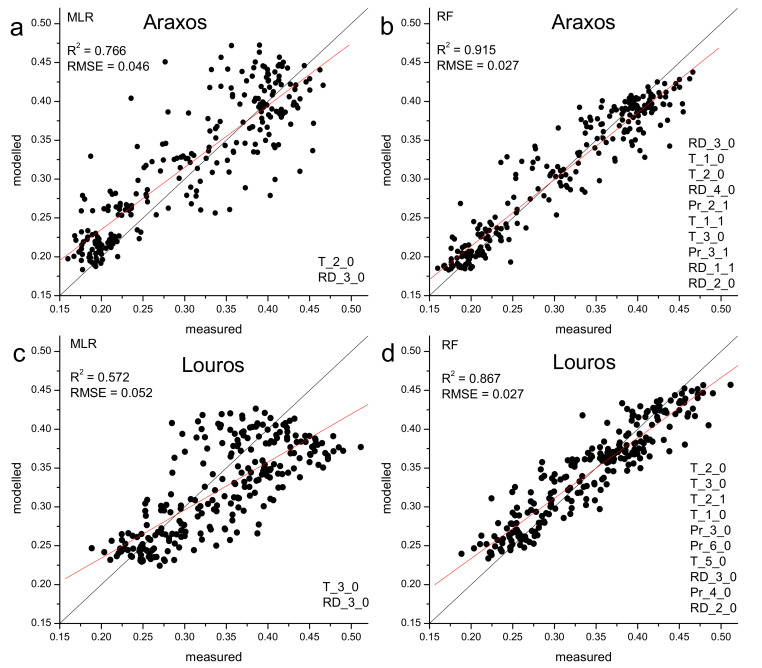
Regressions between measured and modelled EVI through multiple linear regression (MLR, **a**,**c**) and random forest machine learning (RF, **b**,**d**) for Araxos (**a**,**b**) and Louros (**c**,**d**). In multiple linear regressions (**a**,**c**) the two climatic parameters participating in the models are shown in the right lower corner of each graph. In machine learning models (**b**,**d**) the ten most important parameters are shown. In both cases parameter importance decreases from top to bottom. Black lines correspond to the 1:1 lines and red ones to the regression lines. Climatic parameters are described by an acronym for parameter description (T for temperature, Pr for precipitation, and RD for rain days) followed by a number showing the number of the corresponding months and a number corresponding to the lag time, for example T_2_1 refer to temperature of two months before one month.

As shown on Figure 9, EVI may be predicted by similar parameters for both sites through multiple linear regression analysis, i.e., temperature of the previous two months for Araxos and three months for Louros and rain days of the precious three months for both sites. However, the machine learning approach—in which all climatic parameters are included—results in much stronger models compared with the multiple linear regression approach, as judged by R^2^, RMSE and the regression line which is closer to the 1:1 line. It is worth to note, that the parameters determined by the multiple linear regression approach are among the most important ones determined by the machine learning approach, but the inclusion of additional parameters significantly enhances model efficiency.

## 4. Discussion

In this study, the phenological differences between two sites dominated by the semi-deciduous shrub *Phlomis fruticosa*, were examined using MODIS EVI time-series. *P. fruticosa* has been extensively studied from an ecophysiological point of view with both field measurements [33,34] and in combination with satellite data [22,25]. The most important characteristic of its growth pattern is the massive leaf shedding during spring, as an adaptation to the adverse conditions of the hot and dry Mediterranean summer, accompanied by autumn revival after the onset of the autumn rains.

### 4.1. EVI Tntra- and Inter-Annual Fluctuation and Phenology Metrics

The first target of the present study was to monitor seasonal and inter-annual fluctuation of EVI and, subsequently, to identify key phenological events, in order to analyze the temporal dynamics of *P. fruticosa* community in two distinct sites.

The two sites examined in this study are located near to the shoreline of western Greece but have a latitude difference of about 1°. Accordingly, during the 21-year study period, the southern site (Araxos) appeared more xeric compared with the northern site (Louros, Figure 2).

As shown in Figure 3, Figure 4 and Figure 5, satellite data can capture and effectively describe the complex phenology of the semi-deciduous shrub. The inter-annual variability of EVI values is considerable and this is well depicted in two extreme years, the dry 2001 and wet 2016, which are presented in Figure 5 in relation to precipitation. Both sites exhibit an analogous profile concerning the prolonged drought phase (denoted by EVI < 0.3) at the dry year, which is considerably shortened during the wet year. In the relevant remote sensing literature, several studies have reported a detailed single-species phenology monitoring, emphasizing the importance of spatially explicit analyses and the study of phenological trends in small-scale level [15,39,40]. Especially in the fragmented and highly heterogeneous Mediterranean vegetation, this approach connects small- to large-scale information on community or ecosystem function, which would be otherwise lost if only regional level is considered [41].

The timing of certain phenological events that describe the annual cycle of *P. fruticosa* differs in various degrees between the two sites. In the southern Araxos, the earlier spring drop onset, the later autumn revival onset, the prolonged dry period and the earlier appearance of maximum EVI are important in shaping the annual picture and statistically significant. It is well documented that the above-described phenological transitions relate to greening-up or senescence processes which control the community function, state, and productivity [22,25]. Thus, the phenological transitions are influenced and in turn may influence the microclimate, generating gradients of humidity and temperature and affecting topsoil characteristics. Playing such a crucial role in microclimate modification the phenological patterns may have long-term feedback on larger-scale vegetation–climate interactions [2].

### 4.2. Climatic Control on Phenological Events

In semi-arid Mediterranean ecosystems, the high inter-annual variability of EVI suggests that the “memory effects” of previous year’s climate are minimized, as proposed by Catorci et al. [42]. Therefore, our analysis of the relationship between phenological events and climatic drivers was focused on up to 6 months preceding each certain transition. The spring and dry-period-related events were influenced by both precipitation and temperature of a short preceding period. The spring drop of winter leaves indicates the end of winter growth and the “preparation” of *P. fruticosa* to face summer adverse period. Spring precipitation was the major driver of SDO, whereas its duration was influenced by summer precipitation in Louros and summer temperature in the drier Araxos site. Spring phenology has been proved to be sensitive to climatic control in Mediterranean-type ecosystems, possibly due to the high variability of climatic parameters during spring [4,43]. Working with Mediterranean grasslands Catorci et al. [42] reported that rainfall in March and drought stress in April and May were the main drivers of satellite-derived spring biomass production. Climatic parameters linked to moisture control are predominant in shaping vegetation response in Mediterranean, semi-arid and arid ecosystems [44]. Precipitation totals over the preceding three months have been found to correlate with the start of growth season in various Mediterranean regions [4]. Piedallu et al. [45] highlighted the positive correlation of spring temperature with vegetation greenness in an elevation gradient in South France, while stating that rainfall played a minor role in the overall region response, except for the most arid microsites.

The onset of the dry period for *P. fruticosa* was determined as the time-point at which the EVI reaches the 0.3 threshold, denoting a massive leaf loss thus a significant decrease in LAI. Increased spring precipitation retards DPO, but increased spring temperature advances it in Araxos, whereas the temperature of February is the only important parameter in Louros. The duration of dry period and the DPE is influenced mainly by precipitation in both study sites. The seasonal timing of rainfall events is important in determining their effects on phenology [43]. Especially in semi-arid systems and drylands, the precipitation during water-deficit periods is significantly more important in driving phenology than the rain during favorable moisture conditions. Broich et al. [31] suggested that the stronger correlation patterns with single-month compared with multi-month aggregated drivers indicate that rainfall at a specific time-point determines most phenological events. Corroborating this conclusion, our findings suggest that 7 out of 13 phenology metrics were influenced by single or double-month precipitation related parameters in the drier site of Araxos.

The autumn revival of *P. fruticosa* is characterized by the resurrection of summer leaves and the massive production of the winter leaves, thus it is related to an abrupt increase in EVI values. The ARO is primarily driven by the precipitation of the previous 5 months in Louros, whereas in Araxos an equal contribution of the previous 4 months precipitation and the rain days of previous 3 months is recorded. This result is in accordance with Cabello et al. [43], who reported that the earlier arrival of the first rains after the summer adverse period significantly account for the acceleration of growth period onset in Mediterranean drylands. To the same direction was the effect of precipitation on vegetation greening in semi-arid sites of Tunisia [17]. On the contrary, Horion et al. [46] stated that temperature of the last month was the major climatic constraint for growth from the start of vegetative growth to flowering in two studied sites in the Mediterranean basin.

The study of possible climate change effects on phenology was significantly advanced by the use of satellite remote sensing as an efficient tool for continuous vegetation monitoring in large temporal scales [47,48]. The present 21-year study covers an adequate period to evaluate trends in timing of phenological events. A significant trend for earlier spring drop onset in Araxos site was evident following the similar downward trend of April precipitation, which was found to be the most influential climatic factor at this particular event and site. On the contrary, the SDE showed a delay rate of 3.8 days year^−1^, in response to July-August temperature increase. Both SDO advancement and SDE delay resulted in a significant trend for prolonged SDD, at a rate of 4.9 days year^−1^. Spring events in Louros and the other phenophases of *P. fruticosa* showed weak or no trends for change. The advancement of spring phenology is one of the consistent observations across Northern Europe [18], North America [30], and China [5] during the two recent decades. In Mediterranean-type ecosystems, the spring phenology trends show scattered spatial pattern according to a comprehensive study of Ivits et al. [4]; over the southern Mediterranean region, an earlier start-of-season was observed, whereas over parts of the northern Mediterranean basin a growing season shift towards later dates was evident. Concerning the climate forcing of spring phenology trends, March rainfall was reported as the main driver of NDVI variability [42], whereas in accordance with our results Cabelo et al. [43] also reported a trend for reduced spring precipitation which accounted for spring phenology variations. Temperature rising has also well documented consequences in vegetation phenology, especially in semi-arid ecosystems [49,50,51]. An interesting outcome of the analyses presented here is that the contribution of both temperature and precipitation is higher in shaping the Louros phenological profile, whereas the more xeric Araxos depend more on rainfall. This is in accordance with the relevant literature, where a positive link of precipitation and satellite-derived phenology has been generally observed, but a stronger relationship has been reported for xeric compared with colder and wetter areas [45,52].

Temperature and rain days proved to be the main climatic drivers of EVI profile for both Araxos and Louros sites, according to multiple linear regressions and machine learning approach (Figure 9). Specifically, the temperature of the previous two (Araxos) or three (Louros) months and the number of rain days for the preceding three months account for the overall variation of EVI in the 21-year period. The same climatic factors with variable time windows are present in the set of the 10 most influential parameters derived from the machine learning approach, which is increasingly adopted in studies involving time-series. It is interesting to examine these results in the context of climate change. Mediterranean ecosystems are vulnerable due to intense anthropogenic pressure, natural disturbances (i.e., drought and fires), and a highly fluctuating climate, with main characteristic the erratic precipitation patterns [4]. The scenarios of climate change impact on the existing precipitation and temperature regimes include a large reduction in annual precipitation and an increase in inter-annual variability. The latter is expected to result in more heavy rainfall concentrated in fewer rain days, thus prolonged and frequent drought events. Thus, it is crucial to include the parameter of rain days in the studies of climatic forcing on phenology and ecosystem productivity.

The findings of the present work revealed differences in both *P. fruticosa* phenology and its climatic drivers between two sites being only 1° apart with small differences in climate. We may expect even more significant differences between regions with completely different climatic profile, though the direction and magnitude of response cannot be predicted. The single-species sites examined in this work facilitated EVI signal analysis and drawing conclusions but simultaneously may be considered a study’s limitation concerning the applicability of the methodology in more diverse and species-rich ecosystems. Future studies may examine distant Mediterranean sites where significantly different climatic conditions prevail. Moreover, incorporating other key Mediterranean species will be crucial for understanding the dynamics of Mediterranean species phenology in regard to climate. Since *P. fruticosa* is a key species in garrigue formations in Greece (also called phrygana), the findings of the present study may give valuable baseline information for future studies on more complex garrigue ecosystems phenology and the involved climatic drivers.

## 5. Conclusions

The complex phenological cycle of the drought semi-deciduous *P. fruticosa* was clearly depicted in the satellite-derived EVI seasonal fluctuations in both studied sites, the southern Araxos and the northern Louros. The phenology metrics were differentiated between the two sites. The contribution of both temperature and precipitation is higher in shaping the Louros phenological profile, whereas the more xeric Araxos depends more on rainfall. In Araxos, a trend for SDO advancement and more prolonged SDD was recorded during the last 21 years, closely related to certain precipitation and temperature trends. The results of the present study revealed the importance of analyzing the seasonal timing of the phenological events in the lifecycle of a typical species of the Mediterranean ecosystem and of identifying the climatic drivers of their profiles changes. This approach, which focuses on a single species and explicit small-spatial-scale information, will be crucial in connecting small- and large-scale vegetation responses to climate crisis.

## Figures and Tables

**Table 1:** Phenological events derived from the EVI curves, their abbreviations, and characteristics.

Phenological event		Abbreviation	Characteristics
Spring drop	onset	SDO	Day of Year
	end	SDE	Day of Year
	duration	SDD	Number of days. SDD = SDE – SDO
Autumn revival	onset	ARO	Day of Year
	end	ARE	Day of Year
	duration	ARD	Number of days. ARD = ARE – ARO
Dry period	onset	DPO	Day of Year
	end	DPE	Day of Year
	duration	DPD	Number of days. DPD = DPE – DPO
Annual maximum EVI		Max EVI	
Date of Max EVI			Day of Year
Annual minimum EVI		Min EVI	
Date of Min EVI			Day of Year

**Table 2:** Average data (±SD) for the phenological events described in Table 1 for the two study sites, their difference (Araxos–Louros), and the significance of their difference (P, paired *t*-test). DOY, Day of Year; ND, Number of Days.

Phenological Event		Araxos	Louros	Difference	P
Spring Drop	Onset (DOY)	95 ± 14	110 ± 11	−15	<0.001
	End (DOY)	212 ± 33	239 ± 17	−27	0.004
	Duration (ND)	117 ± 38	129 ± 23	−11	0.242
Dry Period	Onset (DOY)	131 ± 8	171 ± 19	−40	<0.001
	End (DOY)	304 ± 33	292 ± 37	11	0.033
	Duration (ND)	172 ± 34	121 ± 47	51	<0.001
Autumn Revival	Onset (DOY)	268 ± 23	255 ± 22	13	0.007
	End (DOY)	345 ± 40	340 ± 42	5	0.486
	Duration (ND)	77 ± 33	85 ± 32	−8	0.238
Max EVI		0.439 ± 0.026	0.473 ± 0.028	−0.034	<0.001
Date of Max EVI	(DOY)	81 ± 17	103 ± 10	−22	<0.001
Min EVI		0.179 ± 0.011	0.226 ± 0.021	−0.048	<0.001
Date of Min EVI	(DOY)	253 ± 23	245 ± 20	8	0.171

## Data Availability

The data is available from the authors upon request.
